# A systematic review and meta-analysis of safety and efficacy of safinamide for motor fluctuations in patients with Parkinson's disease

**DOI:** 10.12688/f1000research.21372.1

**Published:** 2019-12-10

**Authors:** Mohamed Abdelalem Aziz Ahmed

**Affiliations:** 1 School of Advanced Education Research and Accreditation, Universidad Isabel I, Burgos, Spain

**Keywords:** Safinamide, Parkinson's disease, Motor, Dopamine agonist, Movement disorder, UPDRS, Dopamine, Add-on

## Abstract

**Background: **Safinamide, a recently developed drug with several mechanisms of action has been investigated as an add-on therapy for Parkinson's disease patients suffering from motor complications due to the usage of anti-Parkinson's medications such as levodopa and dopaminergic drugs. The aim of the study is to investigate the efficacy and safety of Safinamide as add-on therapy for Parkinson's disease patients.

**Methods: **A computerized literature search was conducted of PubMed, EMBASE, ClinicalTrial.gov and Cochrane Library until August 2019. We selected relevant randomized controlled trials comparing safinamide groups to placebo groups. Relevant outcomes were pooled as mean difference (MD) and risk ratio (RR) using Review Manager 5.3.

**Results:** We found that the overall MD of changes in “off-time” and “on time without troublesome dyskinesia” favored the safinamide group over the placebo group (MD -0.72 h, 95% CI -0.89 to -0.56 and MD 0.71 h, 95% CI 0.52 to 0.90, respectively). Additionally, the overall MD of change in Unified Parkinson's Disease Rating Scale part three (UPDRS III) favored the safinamide group (MD -1.83, 95% CI -2.43 to -1.23). In case of adverse events, the pooled meta-analysis did not favor the safinamide group over the placebo group.

**Conclusions: **In this study, we provide class I evidence about the potential role of safinamide as an add-on therapy for Parkinson's disease patients suffering from motor fluctuations. However, a few included studies did not mention the data of important outcomes. Also, we report high risk of bias in individual studies. Future randomized controlled trials with different doses are recommended to provide more evidence for the efficacy and safety of safinamide as a treatment for motor complications of anti-Parkinson's medications.

## Introduction

Parkinson’s disease prevalence in the fourth decade of life is 41 people per 100,000 and increases to 1,900 people per 100,000 among those who are older than 80 ^1^. According to these statistics, Parkinson’s disease is the second most common neurodegenerative disease after Alzheimer’s disease.

The main pathology of Parkinson’s disease is loss of dopaminergic innervation in the nigrostriatal pathway and spread to various regions in the brain. This loss leads to two types of symptoms; motor and non-motor. Motor symptoms include tremors, rigidity, and bradykinesia. Non-motor symptoms include depression, inability to sustain attention, and sometimes psychosis, especially hallucinations.

Despite the recent medications and updates in the field of pharmacology, there is no definitive treatment that can stop the progress of dopamine receptor loss in the nigrostriatal pathway. Therefore, we only use symptomatic medications for both the motor and non-motor symptoms.

The main symptomatic medications of Parkinson’s disease are Levodopa (L-Dopa) ^2, 3^, dopamine agonists (DAs), and monoamine oxidase-B (MAO-B) inhibitors. Unfortunately, increasing the dose of these medications, especially L-Dopa, leads to motor side effects such as end-of-dose wearing off and dyskinesia, which can be irritating for patients ^4– 6^. Recently, a novel drug called safinamide was developed, which can reduce the side effects of these symptomatic medications, especially motor adverse events.

Safinamide has several dopaminergic and non-dopaminergic mechanisms of action such as sodium channel blockade, calcium channel modulation, and MAO-B inhibition ^7^. The main goal of these mechanisms is inhibiting glutamate release and subsequently, improving motor symptoms ^8^.

Recently published studies discussed the beneficial role of safinamide for treatment of motor complications of Parkinson’s medications ^9– 14^. Some of them suggest that usage of safinamide improves quality of life and delays the motor deterioration of Parkinson’s disease; thus, our study aims to evaluate the safety and efficacy of safinamide use for Parkinson’s patients. According to our knowledge, this is the first meta-analysis that provides class I evidence for the useful usage of Safinamide for Parkinson’s motor complications.

## Methods

The Preferred Reporting Items of Systematic reviews and Meta-Analyses (PRISMA) guidelines were followed during the preparation of this manuscript ^15^. We specified the inclusion criteria, methods of searching, and analysis in advance. The methods and analyses were conducted in strict accordance to the guidelines of the Cochrane Handbook for Systematic Reviews of Interventions and the Methods Guide for Comparative Effectiveness Reviews ^16, 17^.

### Eligibility criteria

Studies that fit all of the following criteria were included in the meta-analysis:

(1) Population: Studies whose population was patients with idiopathic Parkinson’s disease (diagnosed using the UK Parkinson’s Disease Society Brain Bank Criteria) ^18^ and including all stages of Parkinson’s disease (mid to late stages)
(2) Intervention: Studies where patients receive safinamide as an experimental drug (all doses are considered) and continue receiving dopamine agonist treatment
(3) Comparator: Studies where the control group received a placebo
(4) Study design: Studies that were described as prospective randomized controlled trials

Studies were excluded based on the following criteria:

(1) Studies using drugs other than safinamide as experimental drugs
(2) Studies not using safinamide ass add-on therapy for motor fluctuations in Parkinson’s disease
(3) Animal studies, *in vitro* studies, case reports/case series, conference abstracts, or review articles
(4) All studies other than randomized controlled trials (case reports, conference abstracts, and review articles)
(5) Studies unavailable in the English language.

### Information source and literature search

A computer literature search was performed of online databases: PubMed, EMBASE, ClinicalTrial.gov and the Cochrane Library from 1960 to the end of August 2019 (the time of the last search) using the following keywords: (“Safinamide”[All Fields]) AND (“Parkinson disease”[MeSH Terms] OR (“Parkinson”[All Fields] AND “Disease”[All Fields]) OR “Parkinson disease”[All Fields] OR (“Parkinson’s”[All Fields] AND “Disease”[All Fields]) OR “PD”[All Fields]). No restrictions by language or publication period were used.

### Study selection

After removal of duplicate articles, two reviewers (M.H and R.G) screened a spreadsheet of titles and abstracts independently using Microsoft Excel 2013 (windows version). Full text studies selected were examined independently by the same two reviewers, the third reviewer (A.N) solve any disagreement by discussion with the main author before the final selection. The independent reviewers are acknowledged for their generous help in searching, screening, and data extraction processes. We did not need to contact any study investigator for further clarification.

### Data collection process and data items

An online data extraction sheet was constructed. One independent reviewer (M.H) extracted the data from included studies and entries were checked by the main author. The data extraction form included the following domains: 1) study ID; 2) year of publication; 3) country; 4) study design (randomized controlled trials only); 5) follow-up duration; 6) safinamide dose; 7) population definition; 8) inclusion and exclusion criteria; 9) sample size; 10) baseline characteristics; 11) available data of outcome measures (pre, post, and change from baseline); and 12) quality assessment domains. A copy of data extraction form is available as extended data ^19^.

### Risk of bias in individual studies

Cochrane risk of bias assessment tool was used to assess the risk of bias in randomized controlled trials. We assessed the following risks: 1) Selection bias; 2) performance bias; 3) detection bias; 4) attrition bias; 5) reporting bias; and 6) any other source of bias that might have influenced the study data ^20^. One reviewer (A.N) and the main author rated each domain separately as low, high or unclear risk of bias. We used Review Manager software (RevMan 5.3) to summarize the risk of bias of included randomized controlled trials.

### Efficacy measures

The efficacy of drugs treating motor complications in Parkinson’s disease was assessed for the following outcomes:

(1) Unified Parkinson’s Disease Rating Scale part three (motor part) (UPDRS III): The unified Parkinson’s disease rating scale ^21^ is a reliable score of four parts to assess the severity of Parkinson’s symptoms. Part three indicates the motor score, which is the main measure for motor function in Parkinson’s disease patients.
(2) Patient-reported diaries: Patient diaries gave information about the duration of the following motor outcomes: "on time with non-troublesome dyskinesia", which means the duration of absence of dyskinesia associated with the long term usage of Parkinson’s dopaminergic drugs such as levodopa and "off-time", which is the duration of returning motor and non-motor symptoms of Parkinson’s disease, even with the use of levodopa and other antiparkinsonian drugs.
(3) Dyskinesia Rating Scale (DRS): Long term usage of dopaminergic drugs leads to involuntary motor movements. This scale is one of the best scales to assess these motor complications ^22^. It measures the following outcomes: "on time dyskinesia" and "off-time dyskinesia". Additionally, it gives recommendations for descriptions of each type of involuntary movement that can be used when talking with people affected by Parkinson’s.
(4) Clinical Global Impression scale – Severity of Illnes (CGI-S): A seven-point scale used to measure symptom severity, efficacy of the treatments, and treatment response in studies containing patients with mental health issues ^23^.
(5) Unified Parkinson’s Disease Rating Scale part two (UPDRS II): UPDRS is the most commonly used scale to assess the clinical condition of Parkinson’s disease. This is the second part of UPDRS scale, which is used to evaluate the activities of daily life (ADLs) such as hygiene, speech, dressing, and swallowing ^24^.
(6) Parkinson’s Disease Questionnaire (PDQ-39): A self-reported questionnaire with 39 items ^25^. This questionnaire is mainly used to evaluate the difficulties Parkinson’s disease patients face in eight quality of life dimensions, including ADLs, cognition, attention, working memory, depression, social support, social relationship, and functional mobility.
(7) Mini-Mental State Examination (MMSE) scale: A 30-point questionnaire used mainly to evaluate cognitive function. Its usage includes the following: estimating disease progression, severity of impairment of cognitive functions, and documenting the response of mental ill patients to treatment ^26^.
(8) Hamilton Depression Rating Scale (HAM-D): A 21-item test widely used in clinical practice and pharmaceutical trials to assess depressive symptoms ^27^.

### Synthesis of results

Since all the data in the study are continuous data, each efficacy measure is reported as mean difference (MD) between the two groups from the baseline to endpoint, along with its standard error (SE). Both were pooled using the DerSimonian-Laird random effect model. In the case of studies reporting data at multiple time points, the last endpoint was considered.

The overall MD was interpreted with the consideration that efficacy measures are in different directions; an improvement in "on time without troublesome dyskinesia" would be indicated by an increased MD, while an improvement in UPDRS III, “off-time”, UPDRS II, DRS, PDQ-39, MMSE, and HAM-D scores would be indicated by a decreased MD.

The proportion of risk ratio (RR) was used to pool the adverse events reported in the studies to the total number in each group between the two groups in the DerSimonian-Laird random effect model. To examine heterogeneity of studies, forest plot visual inspection was used and assessed using the Cochrane Q and I2 tests using RevMan version 5.3 for windows.

### Calculation of missing data

A few studies, such as as Stoochi (2004), did not report the MD between the safinamide group and placebo group so it was calculated using the following calculation: [MD = MD experimental – MD placebo]. Standard error was calculated from the standard deviation [standard error = standard deviation⁄√ *n*], 95% confidence interval [(upper limit – lower limit) ⁄3.92], or 90% CI [(upper limit − lower limit) ⁄3.29]. For studies and groups with a sample size of less than 60 patients, the numbers (3.92 and 3.29) were substituted by a value from the table of *t* distributions with degrees of freedom equal to the group sample size minus one.

### Risk of bias across studies

Funnel plots was used to explore the publication bias across studies and to show the relationship between effect size and precision. The evidence of publication bias was assessed using the following: 1) Egger’s regression test, and 2) the Begg and Mazumdar rank correlation test (Kendall’s tau).

### Software

We performed all the analysis and calculations in this meta-analysis using Review Manager software version 5.3 (RevMan 5.3).

## Results

### Study selection

The literature search resulted in 160 studies. After the complete screening process of titles, abstracts, and full texts, 154 studies did not meet the eligibility criteria and six articles with six randomized controlled trials remained with a total of (2556) patients included in the meta-analysis ^9– 14^.

**Figure 1.**
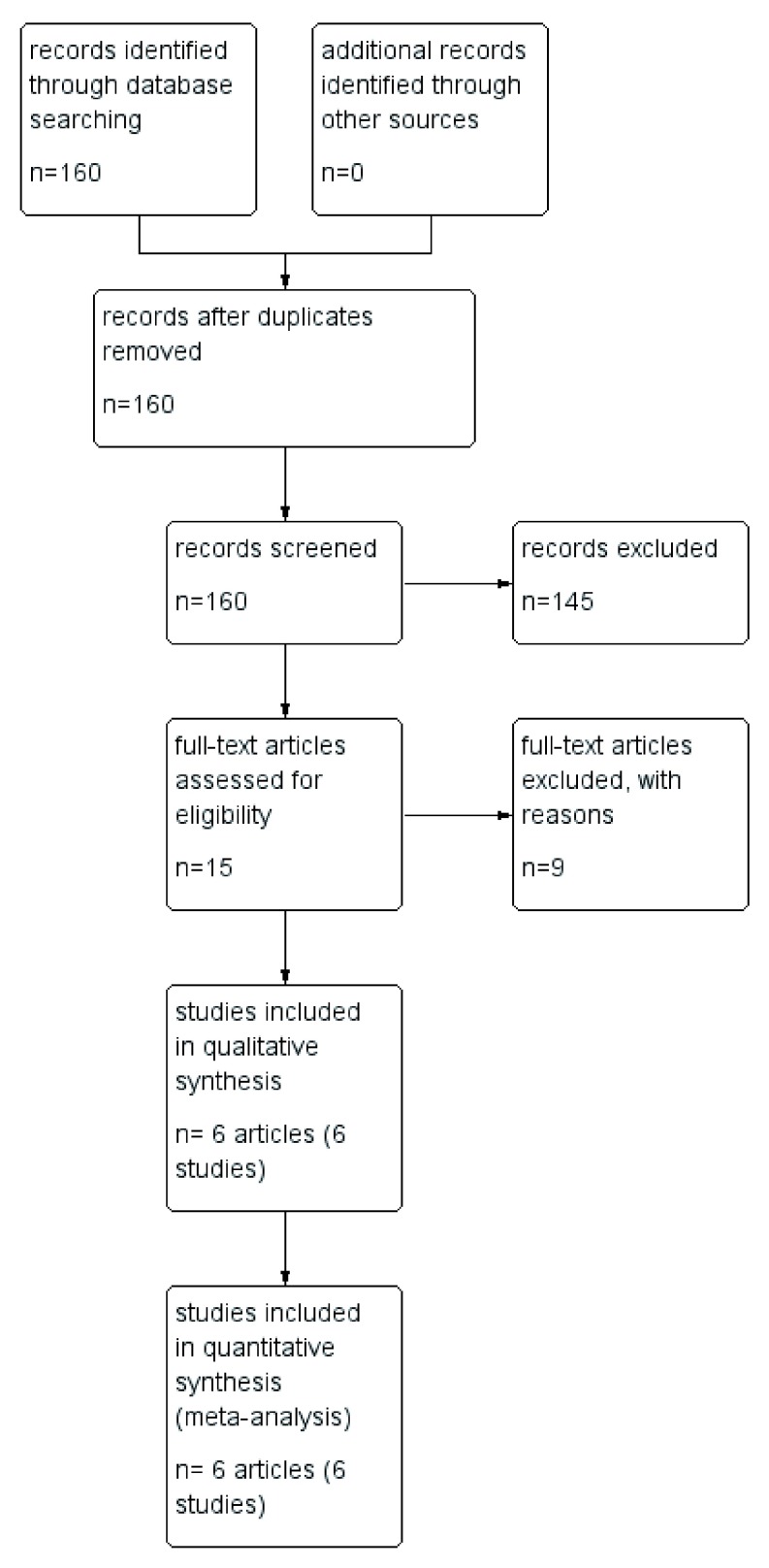
PRISMA flow diagram of the screening and selection for the study.

A description of the flow of study selection is shown in the PRISMA flow diagram in Figure 1.

### Study characteristics

The follow up duration in the studies ranged from 12 weeks in Stoochi *et al.* (2004) and Stoochi *et al.* (2011) ^9, 10^ to 24 weeks in the study by Schapira *et al.* (2012), Borgohain *et al.* (2013), Schapira *et al.* (2016), and Borgohain *et al.* (2014) ^11– 14^.

The daily doses of safinamide received in the studies included in the meta-analysis ranged from 40mg in the study by Stoochi *et al.* (2004) ^9^ to 200mg in studies by Stoochi *et al.* (2011) ^10^ and Schapira *et al.* (2012) ^11^. The population of all studies was homogenous and remained on the dopaminergic treatment during the entire study period.

All patients enrolled in the studies were diagnosed with Parkinson’s disease according to UK Parkinson’s Disease Society Brain Bank Criteria. The criteria of patients excluded from studies were: 1) history of psychiatric disorders, 2) severe and progressive medical illness, 3) patients with dementia, 4) severe dyskinesia. Summary and baseline characteristics of populations of these studies are shown in Table 1.

**Table 1.** Baseline characteristics of the study population in the included studies.

Study ID	Design	Final endpoint	Group	N	Age *	Male %	UPDRS III *	CGI-S *	MMSE *	HAM-D *
Stoochi *et al.* 2011	RCT	12 weeks	Safinamide 200 mg/day	89	58.5 (11.7)	61	19.3 (9.80)	3.1 (0.85)	28.3 (1.54)	4.2 (3.11)
			Safinamide 100 mg/day	90	56.5 (11.3)	66	22.0 (10.15)	3.1 (0.79)	28.9 (1.21)	4.0 (3.43)
			placebo	90	57.3 (10.8)	62	20.7 (9.63)	3.1 (0.76)	28.4 (1.56)	4.3 (3.22)
Schapira *et al.* 2012	RCT	24 weeks	Safinamide 200 mg/day	69	56.5 (25.5)	62.3	20.1 (10.44)	NR	28.3 (1.57)	4.1 (3.08)
			Safinamide 100 mg/day	80	53 (23)	67.5	22.5 (9.28)	NR	29.0 (1.20)	4.1 (3.50)
			placebo	78	55.5 (19.5)	47	21.0 (9.73)	NR	28.3 (1.53)	4.4 (3.14)
Borgohain *et al.* 2013	RCT	24 weeks	Safinamide 100 mg/day	224	60.1 (9.19)	72.8	28.3 (13.30)	4.0 (0.72)	NR	6.0 (3.54)
			Safinamide 50 mg/day	223	60.1 (9.65)	70.4	27.3 (12.66)	4.0 (0.70)	NR	6.0 (3.70)
			Placebo	222	59.4 (9.41)	72.1	28.7 (12.02)	4.0 (0.66)	NR	5.9 (3.70)
Schapira *et al.* 2016	RCT	24 weeks	Safinamide 100 mg/day	274	61.7 (9.0)	62.4	22.4 (11.8)	3 (1.1)	28.7 (1.5)	4.7 (4.0)
			placebo	163	62.1 (8.9)	59.3	23.4 (12.9)	3 (1.1)	28.6 (1.6)	5.0 (4.1)
Stoochi *et al.* 2004	RCT	12 weeks	Safinamide 90 mg /day	34	45.3 (18.9)	NR	16.9 (7.4)	NR	NR	NR
			Safinamide 40 mg/day	33	45 (19.1)	NR	17.6 (7.5)	NR	NR	NR
			placebo	34	45.3 (18.9)	NR	17.1 (8.6)	NR	NR	NR
Borgohain *et al.* 2014	RCT	24 weeks	Safinamide 50 mg/day	223	43 (19.3)	70.4	27.3 (12.66)	NR	NR	5.3 (3.75)
			Safinamide 100 mg/day	224	45 (20.1)	72.8	28.3 (13.30)	NR	NR	5.0 (3.43)
			Placebo	222	42 (18.9)	72.1	28.7 (12.02)	NR	NR	5.5 (4.01)

*Continuous outcomes presented as mean (SD). RCT, randomized controlled trial; UPDRS III; Unified Parkinson’s Disease Rating Scale part three; CGI-S, Clinical Global Impression scale – Severity of Illness; MMSE, Mini-Mental State Examination; HAM-D, Hamilton Depression Rating Scale; NR, not reported.

### Risk of bias within studies

**Figure 2.**
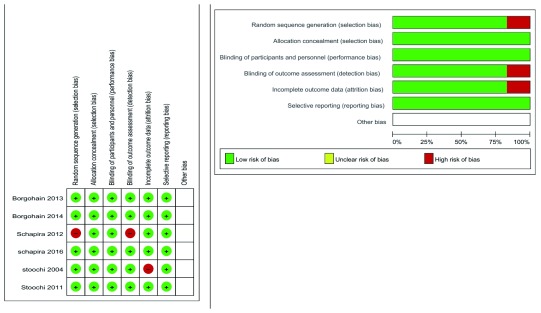
Summary of risk bias according to Cochrane Risk of Bias assessment tool.

The Cochrane risk of bias assessment tool was used to assess the quality of included studies. All included studies had a low risk of bias in terms of random sequence generation, allocation concealment, blinding of participants and personnel, blinding of outcome assessment, incomplete outcome data, and selective reporting except Schapira *et al.* (2012) ^11^, which had a high risk of bias for random sequence generation as the randomization method was not reported and for the blinding of outcome assessment as the method of blinding was not reported, and Stoochi *et al.* (2004) ^9^, which had a high risk for incomplete outcome data because there was no intention to treat analysis for missed or withdrawn patients mentioned in the study. The summary of risk of bias domains is shown in Figure 2.

### Drug efficacy

**Figure 3.**
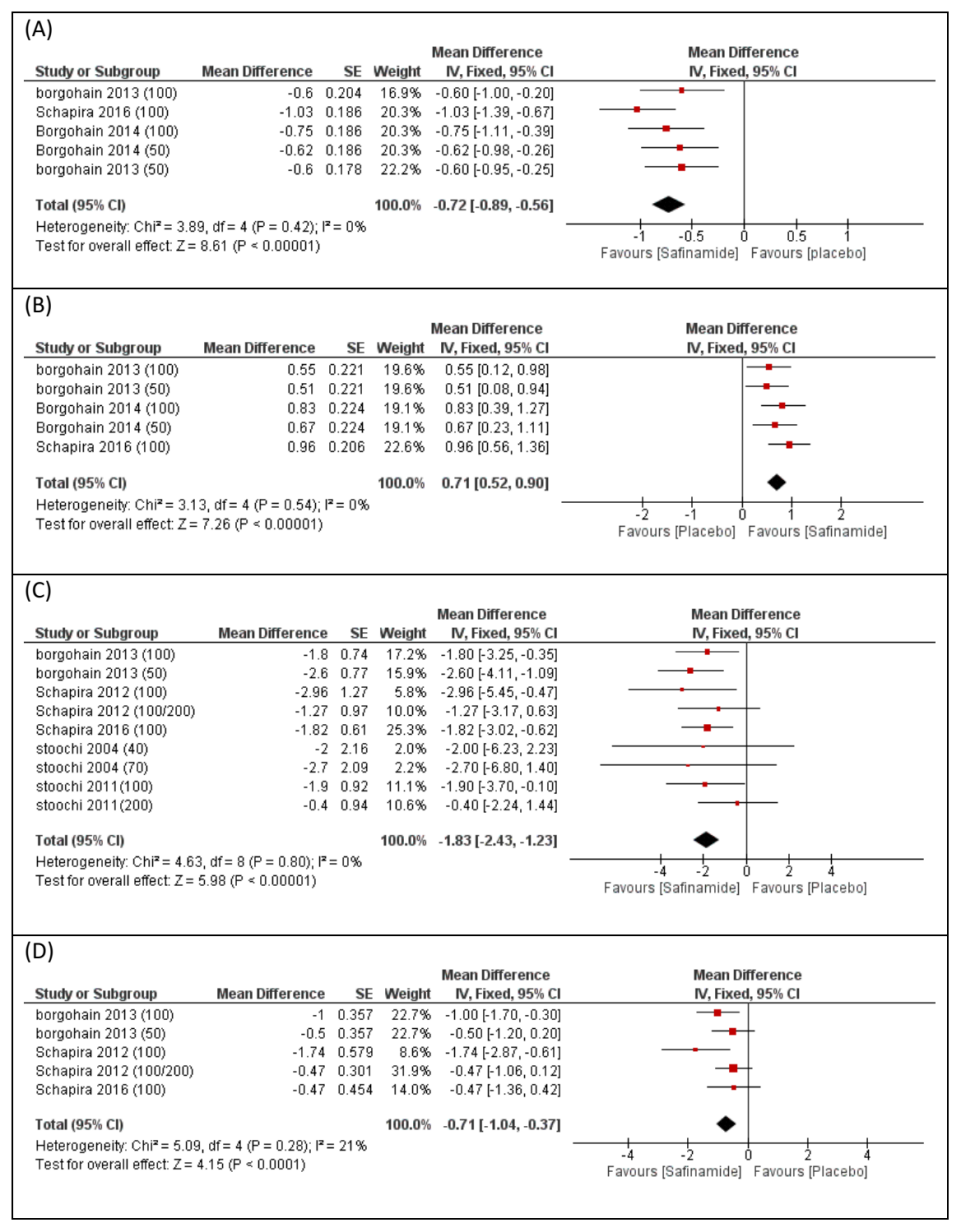
Forest plot of the mean difference and 95% confidence interval of the following outcome; ( **A**) Off time, ( **B**) On-time without troublesome dyskinesia, ( **C**) UPDRS-III, ( **D**) UPDRS-II.

***Off-time.*** The overall MD between the two groups from baseline to endpoint in terms of change in "off-time" favored safinamide over placebo (MD -0.72 h, 95% CI [-0.89 to -0.56], Figure 3A). Pooled studies were homogenous (P=0.42).

***On time without troublesome dyskinesia.*** The overall MD between the two groups from baseline to endpoint in terms of change in "on time without troublesome dyskinesia" favored safinamide over placebo (MD 0.71 h, 95% CI [0.52 to 0.90], Figure 3B). Pooled studies were homogenous (P=0.54).

***UPDRS III.*** The overall MD between the two groups from baseline to endpoint in terms of change in "UPDRS III" favored safinamide over placebo (MD -1.83, 95% CI [-2.43 to -1.23], Figure 3C). Pooled studies were homogenous (P=0.80).

***UPDRS II.*** The overall MD between the two groups from baseline to endpoint in terms of change in "UPDRS II" favored safinamide over placebo (MD -0.69, 95% CI [-1.03 to -0.36], Figure 3D). Pooled studies were homogenous (P=0.26).

**Figure 4.**
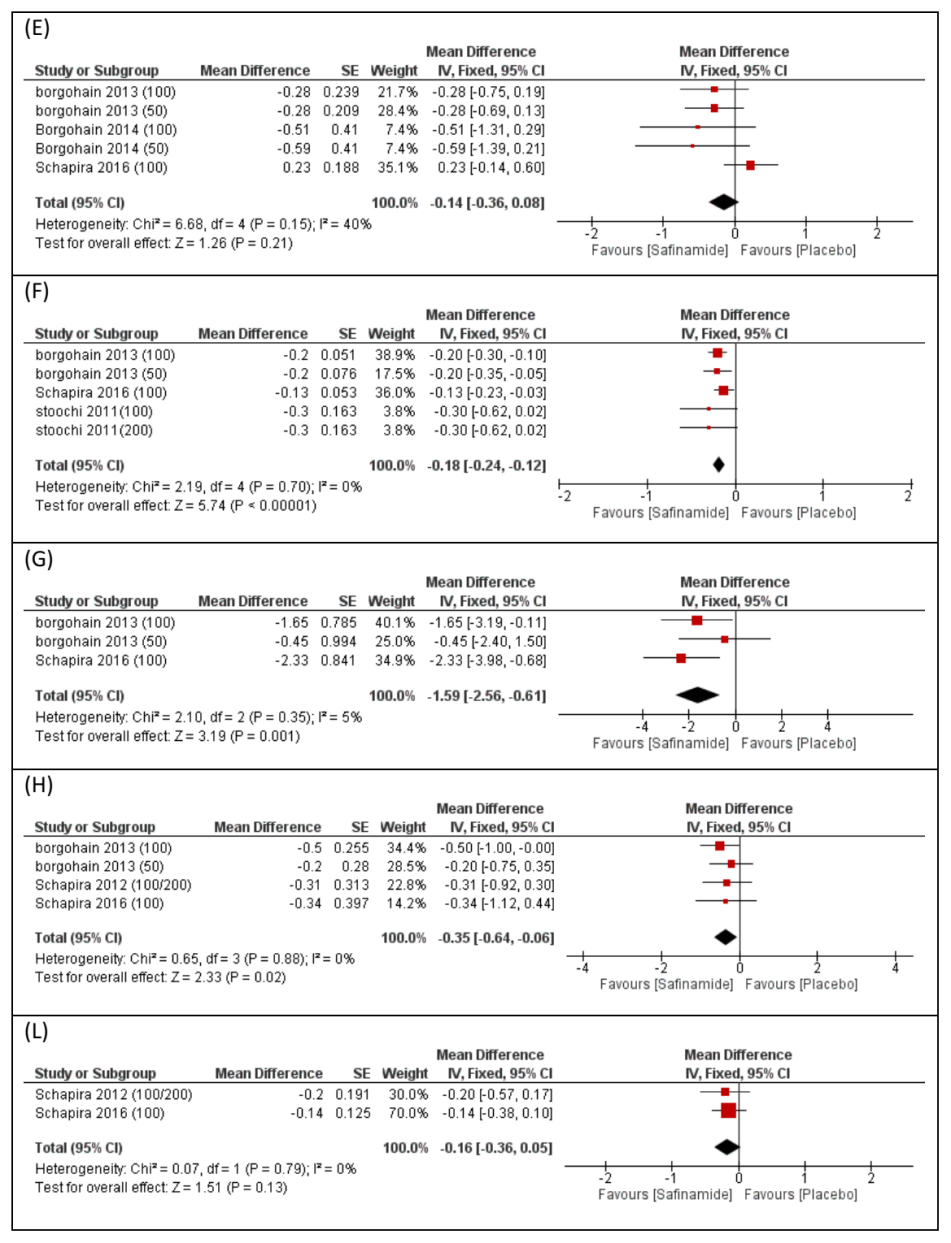
Forest plot of the mean difference and 95% confidence interval of the following outcomes; ( **E**) DRS, ( **F**) CGI-S, ( **G**) PDQ-39, ( **H**) HAM-D, ( **L**) MMSE.

***DRS score.*** The overall MD between the two groups from baseline to endpoint in terms of change in "DRS score" did not favor either of the two groups (MD -0.14 h, 95% CI [-0.36 to 0.08], Figure 4E). Pooled studies were homogenous (P=0.15).

***CGI severity.*** The overall MD between the two groups from baseline to endpoint in terms of change in "CGI severity" favored safinamide over placebo (MD -0.18 h, 95% CI [-0.24 to -0.12], Figure 4F). Pooled studies were homogenous (P=0.70).

***PDQ-39.*** The overall MD between the two groups from baseline to endpoint in terms of change in "PDQ-39" favored safinamide over placebo (MD -1.59 h, 95% CI [-2.56 to -0.61], Figure 4G). Pooled studies were homogenous (P=0.35).

***HAM-D.*** The overall MD between the two groups from baseline to endpoint in terms of change in "HAM-D" favored safinamide over placebo (MD -0.35 h, 95% CI [-0.64 to -0.06], Figure 4H). Pooled studies were homogenous (P=0.88).

***MMSE.*** The overall mean difference between the two groups from baseline to endpoint in terms of change in "MMSE" favored safinamide over placebo (MD -0.16 h, 95% CI [-0.36 to -0.05], Figure 4L). Pooled studies were homogenous (P=0.79).

***Adverse events.*** The following adverse events were reported in the included studies: Back pain, cataeacts, dizziness, hypertension, dyskinesia, headaches, and worsening of Parkinson’s disease, as well as discontinuation due to treatment emergent adverse events (TEAEs), including serious TEAEs, serious drug-related TEAEs, and any TEAEs.

(A) Back pain

**Figure 5.**
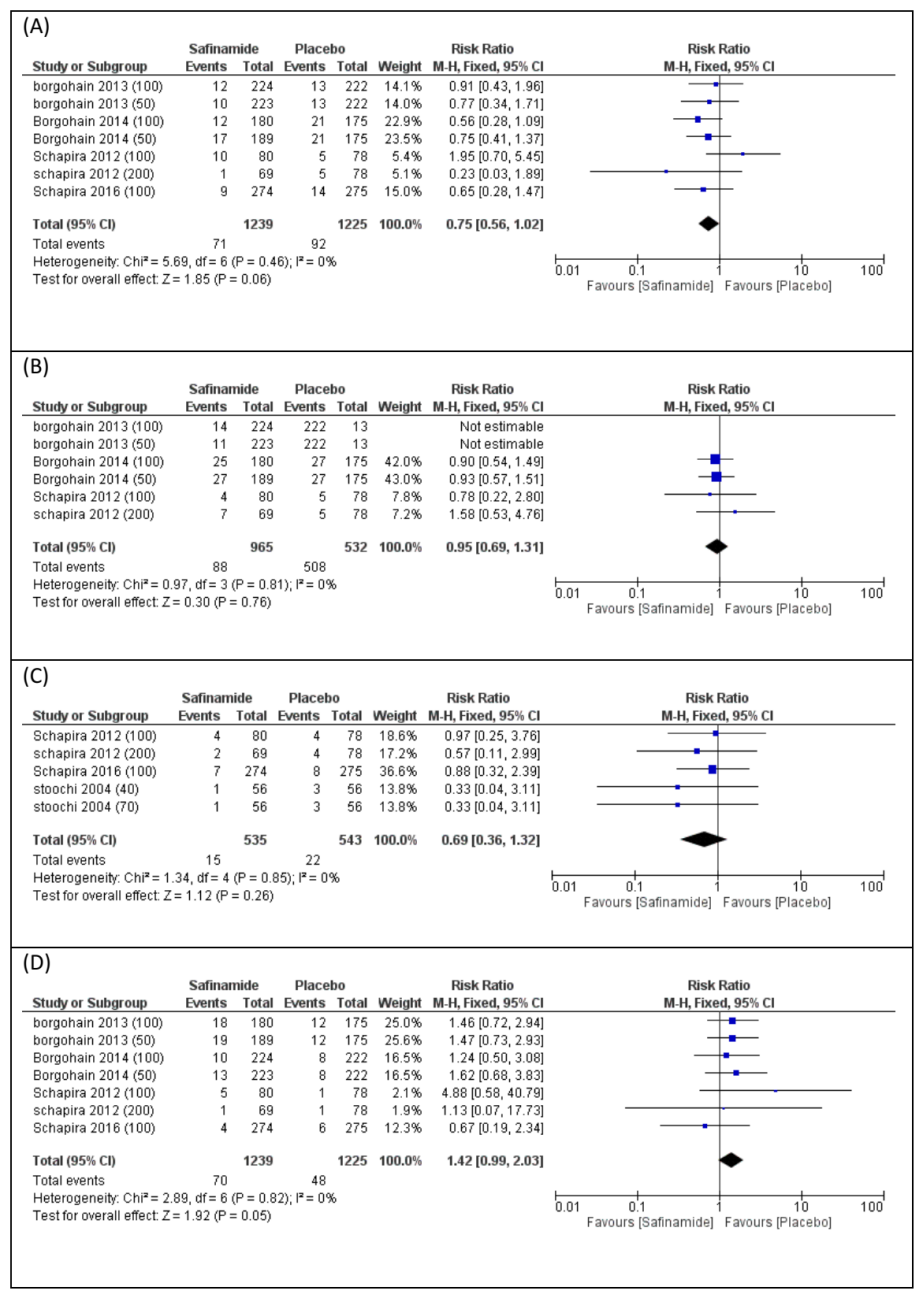
Forest plot presentation of Meta-Analysis for the Following Adverse Events of Safinamide; ( **A**) Back pain, ( **B**) Cataract, ( **C**) Dizziness, and ( **D**) Hypertension.

Seven studies reported back pain. The pooled meta-analysis did not favor either of the two groups (RR 0.75, 95% CI [0.56 to 1.02], Figure 5A). Pooled studies were homogenous (P=0.46).

(B) Cataracts

Six studies reported cataracts. The pooled meta-analysis did not favor either of the two groups (RR 0.95, 95% CI [0.69 to 1.31], Figure 5B). Pooled studies were homogenous (P=0.81).

(C) Dizziness

Five studies reported dizziness. The pooled meta-analysis did not favor either of the two groups (RR 0.69, 95% CI [0.36 to 1.32], Figure 5C). Pooled studies were homogenous (P=0.85).

(D) Hypertension

Seven studies reported hypertension. The pooled meta-analysis did not favor either of the two groups (RR 1.42, 95% CI [0.99 to 2.03], Figure 5D). Pooled studies were homogenous (P=0.82).

(E) Dyskinesia

**Figure 6.**
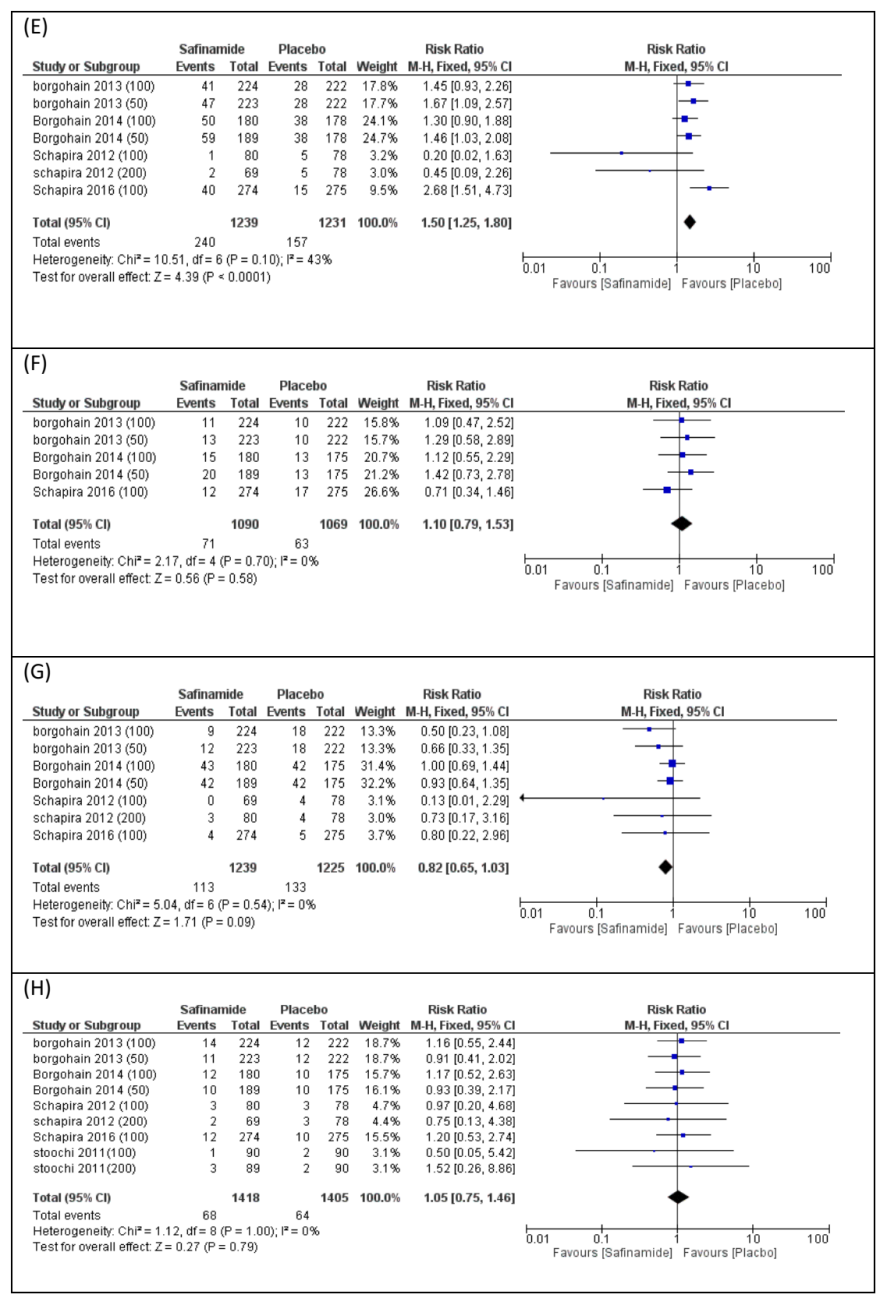
Forest plot presentation of Meta-Analysis for the Following Adverse Events of Safinamide; ( **E**) Dyskinesia, ( **F**) Headache, ( **G**) Worsening Parkinson’s disease, and ( **H**) TEAE’s leads to discontinuation.

Seven studies reported dyskinesia. The pooled meta-analysis showed increase of dyskinesia in patients receiving placebo compared to safinamide (RR 1.50, 95% CI [1.25 to 1.80], Figure 6E). Pooled studies were homogenous (P=0.10).

(F) Headache

Five studies reported headaches. The pooled meta-analysis did not favor either of the two groups (RR 1.10, 95% CI [0.79 to 1.53], Figure 6F). Pooled studies were homogenous (P=0.70).

(G) Worsening Parkinson’s disease

Seven studies reported patients with worsening of Parkinson’s disease during the study. The pooled meta-analysis did not favor either of the two groups (RR 0.82, 95% CI [0.65 to 1.03], Figure 6G). Pooled studies were homogenous (P=0.54).

(H) TEAEs leading to discontinuation

Nine studies reported the number of patients with TEAEs leading to discontinuation of the study. The pooled meta-analysis did not favor either of the two groups (RR 1.05, 95% CI [0.75 to 1.46], Figure 6H). Pooled studies were homogenous (P=1.00).

(I) Serious drug-related TEAEs

**Figure 7.**
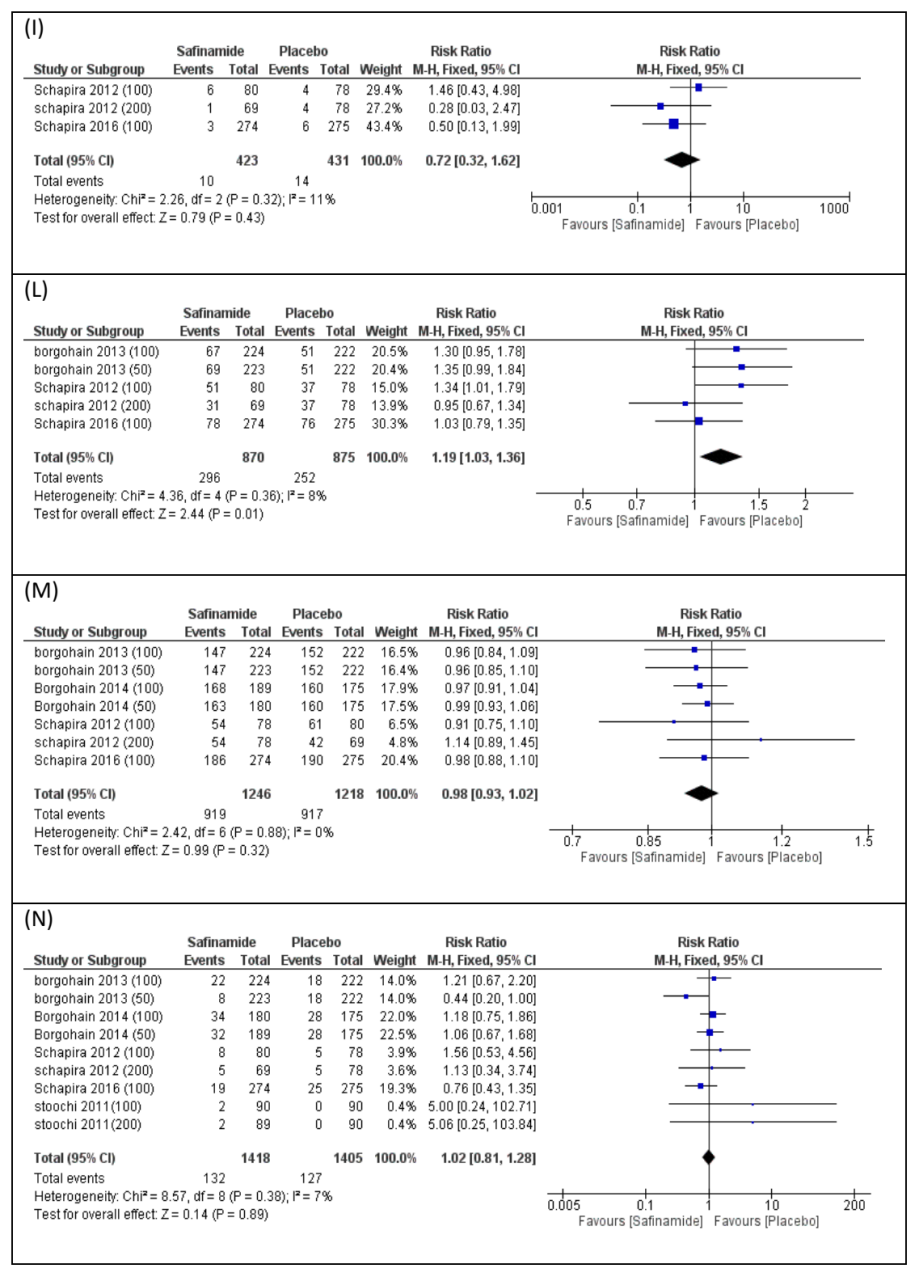
Forest plot presentation of Meta-Analysis for the Following Adverse Events of Safinamide; ( **I**) serious drug related TEAE’s, ( **L**) any study drug related TEAE’s, ( **M**) any TEAE’s, and ( **N**) Serious TEAE’s.

Three studies reported the number of patients with serious drug-related adverse events. The pooled meta-analysis did not favor either of the two groups (RR 0.72, 95% CI [0.32 to 1.62], Figure 7I). Pooled studies were homogenous (P=0.32).

(L) Any drug-related TEAEs

Five studies reported the number of patients with any drug-related TEAEs. The pooled meta-analysis showed an increase in the number of patients with any drug-related adverse events in the placebo group compared to the safinamide group (RR 1.19, 95% CI [1.03 to 1.36], Figure 7L). Pooled studies were homogenous (P=0.36).

(M) Any TEAEs

Seven studies reported the number of the patients with any TEAEs. The pooled meta-analysis did not favor either of the two groups (RR 0.98, 95% CI [0.93 to 1.02], Figure 7M). Pooled studies were homogenous (P=0.88).

(N) Serious TEAEs

Nine studies reported the number of the patients with serious TEAEs. The pooled meta-analysis did not favor either of the two groups (RR 1.02, 95% CI [0.81 to 1.28], Figure 7N). Pooled studies were homogenous (P=0.38).

### Risk of bias across studies

**Figure 8.**
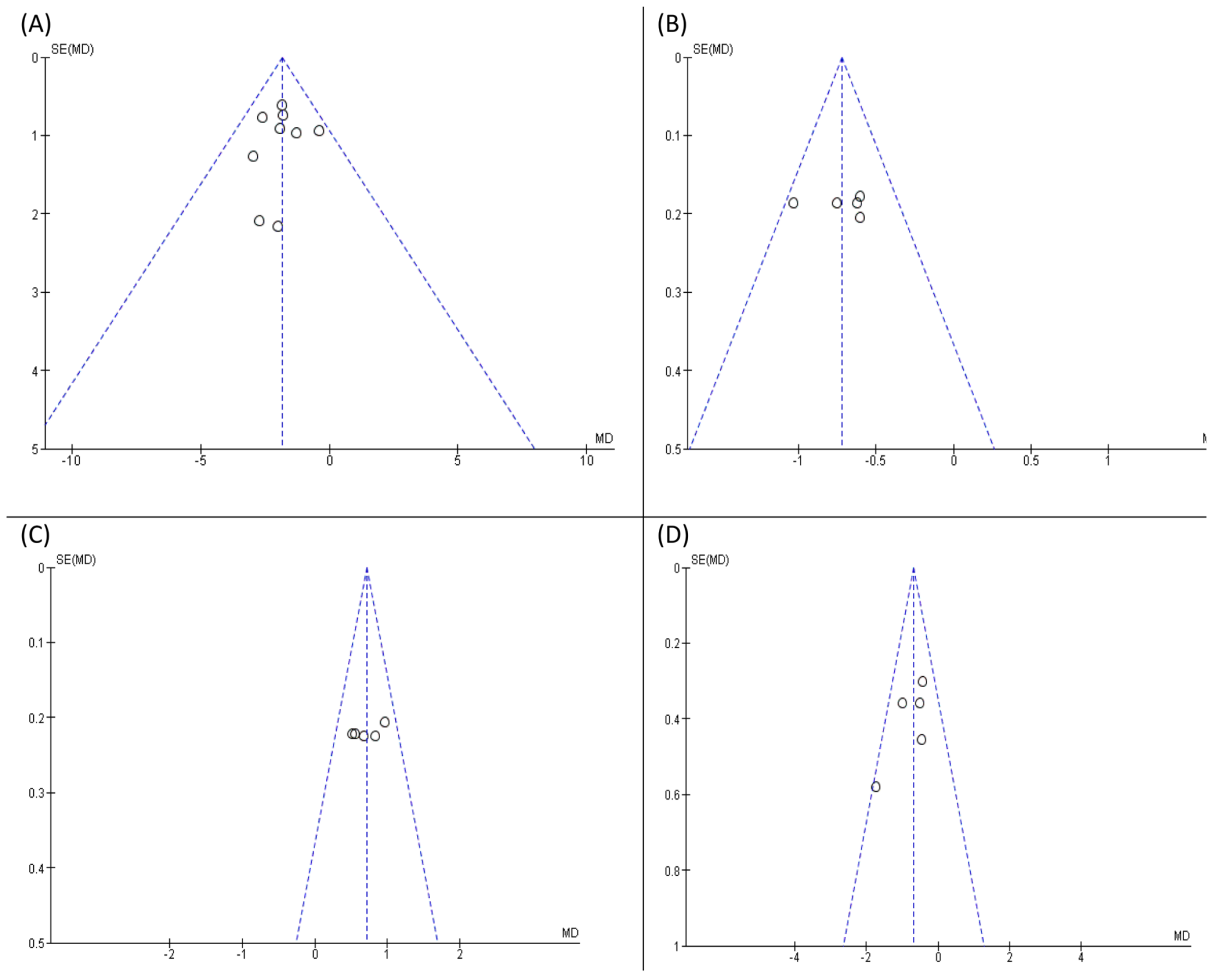
Funnel plots for publication bias for ( **A**) UPDRS III, ( **B**) Off-time, ( **C**) on-time without troublesome dyskinesia, and ( **D**) UPDRS II.

As showed in Figure 8, funnel plots of UPDRS III, off-time, on-time without troublesome dyskinesia, and UPDRS II show no significant publication bias across studies.

## Discussion

### Summary of evidence

The pooled meta-analysis of six studies provides a class I evidence that using safinamide as add-on therapy for Parkinson’s disease is very effective and well tolerated. The meta-analysis shows that safinamide improves motor fluctuations, which is a main side effect of anti-Parkinson’s medications, as reported by patient diaries and measured by "on time without troublesome dyskinesia", "off-time", and UPDRS III score. This novel drug is also improving the quality of life of Parkinson’s disease patients, as measured by the UPDRS II scale, the PDQ-39 questionnaire, HAM-D, and MMSE.

Regarding tolerability, safinamide is a well-tolerated drug and despite increasing the risk of some adverse events such as dyskinesia, which was higher in the safinamide group than the placebo ^10– 14^, the pooled meta-analysis of RRs of adverse events did not show any statistical significance between the two groups of comparison.

### Previous studies

The results obtained from the meta-analysis are consistent with the results of the previous randomized controlled trials in terms of outcomes measuring motor fluctuations and quality of life. “On time without troublesome dyskinesia” and “off-time” are the main outcomes to evaluate motor fluctuations and were mentioned in Schapira *et al.* (2016) with (100 mg daily dose), Borgohain *et al.* (2013 and 2014) with doses of 50 and 100 mg daily. In all the previously mentioned studies, “on time without troublesome dyskinesia” favored the safinamide group over the placebo group ^12– 14^. In addition, the mean difference of “off-time” in all studies favored the safinamide group and this result was consistent with the pooled meta-analysis of included studies ^12– 14^.

The UPDRS III is a very important scale for evaluating the motor symptoms in Parkinson’s patients. Despite the results of Stoochi *et al.* (2011) (200mg daily dose), Stoochi *et al.* (2004) (40mg and 70mg daily doses), and Schapira *et al.* (2012) (pooled doses of 100mg and 200mg daily doses) ^9– 11^, which showed no statistical significance between safinamide and placebo groups, the pooled meta-analysis showed that the UPDRS III score favors safinamide over placebo. The results of the DRS score in the included studies did not favor safinamide over placebo and the pooled meta-analysis did not favor one group either.

Quality of life in Parkinson’s disease patients is measured by UPDRS II, PDQ-39, HAM-D, and MMSE scores. The studies that mentioned the outcomes of quality of life showed results consistent with the pooled meta-analysis that the use of safinamide is preferable to placebo ^10, 11, 14^, except MMSE scores which were mentioned in Schapira *et al.* (2016), and Schapira *et al.* (2012) ^11, 14^ and showed no statistical significance between safinamide and placebo in both the included studies and the pooled meta-analysis.

### Strengths of the study

The strengths of the meta-analysis are the following: 1) multiple search engines were searched and all the possible sources of studies to be included were covered; 2) clear eligibility criteria were provided; 3) multiple reviewers revised every step to ensure accuracy; 4) during the preparation of this manuscript, the PRISMA guidelines were followed; 5) the study was conducted according to the guidelines of the Cochrane Handbook for Systematic Reviews of Interventions in a strict way; 6) the randomized controlled trials included data of high validity and acceptable quality, as indicated by the risk of bias assessment.

### Limitations of the study

The meta-analysis limitations are the following: A) some studies, such as as Stoochi *et al.* (2004), Stoochi *et al.* (2011), and Schapira *et al.* (2012) ^9– 11^ did not mention outcomes such as “on-time without troublesome dyskinesia” and “off-time”, which are important measurements for motor symptoms evaluation; B) there was no standardization in the reporting of adverse events in the included studies; C) there was a high risk of bias in some studies, namely as Stoochi *et al.* (2004) ^9^ and Schapira *et al.* (2012) ^11^.

### Implications for future research

Based on the results of the study, future randomized controlled trials with different doses are recommended to investigate the efficacy of safinamide for Parkinson’s disease patients with motor fluctuations as a side effect of anti-Parkinson’s medications.

## Conclusions

Despite the evidence provided by this meta-analysis, demonstrating the efficacy of safinamide as add-on therapy for treatment of motor complications of anti-Parkinson’s disease medications, future studies are still needed to confirm the safety and efficacy of this novel drug.

## Data availability

### Underlying data

All data underlying the results are available as part of the article and no additional source data are required.

### Extended data

Open Science Framework: A systematic review and meta-analysis of safety and efficacy of Safinamide for motor fluctuations in patients with Parkinson’s disease. https://doi.org/10.17605/OSF.IO/T6H9J ^19^

This project contains the following extended data:

- Data extraction form.xlsx
- Spreadsheets in .xlsx format containing extracted data for drug efficacy outcomes including adverse events

### Reporting guidelines

Open Science Framework: A systematic review and meta-analysis of safety and efficacy of Safinamide for motor fluctuations in patients with Parkinson’s disease. https://doi.org/10.17605/OSF.IO/T6H9J ^19^

Data are available under the terms of the Creative Commons Zero "No rights reserved" data waiver (CC0 1.0 Public domain dedication).
