# Supplementary material for: A systematic review and meta-analysis of safety and efficacy of safinamide for motor fluctuations in patients with Parkinson's disease
Source: F1000Res. 2019 Dec 10;8:2078. [Version 1] doi: 10.12688/f1000research.21372.1 (PMC7205357; doi:10.12688/f1000research.21372.1)
Supplement: Peer review history

# Peer review: Reviewer response for version 1

**DOI:** 10.5256/f1000research.23538.r58806

**Published:** 2020-05-06

**Authors:** Ahmed Negida

**Affiliations:** 1 School of Pharmacy and Biomedical Sciences, University of Portsmouth, Portsmouth, UK; 2 Zagazig University Hospitals, Zagazig University, Zagazig, Egypt

The article describes a systematic review and meta-analysis of published studies on the Safenamide for the treatment of motor fluctuations in Parkinson's Disease. 

 First, the topic is interesting because currently, there is an unmet clinical need to find potential agents that decrease the motor fluctuations and the LED in PD midstage and late-stage PD. 

 Second, the methodology is clear and in accordance with the Cochrane handbook guidelines, while the manuscript is well-written in compliance with the standard reporting guidelines (PRISMA statement) - endorsed by the ICMJEs.

 The statistical analysis was properly done and the data analysis interpretation and discussion are sufficient.

 I do not find any major concerns in the article; I believe it worth the publication. No further changes are required from my point of view.

Are the rationale for, and objectives of, the Systematic Review clearly stated?

Yes

Is the statistical analysis and its interpretation appropriate?

Yes

Are sufficient details of the methods and analysis provided to allow replication by others?

Yes

Are the conclusions drawn adequately supported by the results presented in the review?

Yes

Reviewer Expertise:

I'm a medical doctor, holding an MD degree, and currently doing a Ph.D. in Parkinson's Disease treatment (the same scope of this manuscript). I have several similar publications in the field.

I confirm that I have read this submission and believe that I have an appropriate level of expertise to confirm that it is of an acceptable scientific standard.

# Peer review: Reviewer response for version 1

**DOI:** 10.5256/f1000research.23538.r61383

**Published:** 2020-04-27

**Authors:** Rupam Borgohain, Rukmini Kandadai

**Affiliations:** 1 Department of Neurology, Nizam's Institute of Medical Sciences, Hyderabad, India

The authors published a systematic review and meta-analysis of safety and efficacy of safinamide for motor fluctuations in patients with Parkinson's disease.

 The meta-analysis is the first one and has been conducted well. It demonstrates the efficacy and safety of safinamide in Parkinson's disease and its value in patients with dyskinesias.

 Safinamide can be used in early PD as a single drug or in combination with dopamine agonist. It can be an add on in mid-late PD for motor fluctuations.

 The methodology states that the studies which did not use safinamide as an add on therapy for motor fluctuations in PD would exclude some studies included. 

 However, the studies included in the meta-analysis include patients with early PD as well as those with mid-late PD.

 Early PD patients do not have motor fluctuations, dyskinesias, are less likely to have depression, cognitive impairment and thus this subset may undervalue the actual effect of safinamide.

Are the rationale for, and objectives of, the Systematic Review clearly stated?

Partly

Is the statistical analysis and its interpretation appropriate?

Partly

Are sufficient details of the methods and analysis provided to allow replication by others?

Partly

Are the conclusions drawn adequately supported by the results presented in the review?

Partly

Reviewer Expertise:

Movement disorders, Botulinum toxin, Deep brain stimulation

We confirm that we have read this submission and believe that we have an appropriate level of expertise to confirm that it is of an acceptable scientific standard.

# Peer review: Reviewer response for version 1

**DOI:** 10.5256/f1000research.23538.r61382

**Published:** 2020-03-24

**Authors:** Fabrizio Stocchi

**Affiliations:** 1 Center for Parkinson's Disease, IRCCS San Raffaele Pisana, Rome, Italy

The authors published a systematic review and meta-analysis of safety and efficacy of safinamide for motor fluctuations in patients with Parkinson's disease. There are other review on safinamide but the meta-analysis can be interested. However the paper must be revised addressing some important issues:

 Introduction:

- Non-motor symptoms should be better addressed and ref. implemented and revised.
- Within the other treatment, COMTI are not mentioned

The meta-analysis is well conducted and gave a number of good information, but there are some problems: 

- The studies were grouped according to the presence of the end-point. However for UPDRS III studies with different population were grouped together, moreover in some of these studies UPDR III was the primary end-point but for the other studies it was a secondary end-point. UPDRS scored in an early population give information about the effect of the drug on symptoms, in fluctuators it does not.
- Other parameter were influenced by the dose in the original study (PDQ39, UPDRS II) but putting all the patents together this effect does not emerge giving the false impression that the drug is always efficacious. These aspects should be carefully addressed and discussed.

Are the rationale for, and objectives of, the Systematic Review clearly stated?

Partly

Is the statistical analysis and its interpretation appropriate?

Partly

Are sufficient details of the methods and analysis provided to allow replication by others?

Partly

Are the conclusions drawn adequately supported by the results presented in the review?

Partly

Reviewer Expertise:

Movement disorders, Parkinson’s disease, neuropharmacology, clinical trials.

I confirm that I have read this submission and believe that I have an appropriate level of expertise to confirm that it is of an acceptable scientific standard, however I have significant reservations, as outlined above.
